# Estimating the Amount of the Wild *Artemisia annua* in China Based on the MaxEnt Model and Spatio-Temporal Kriging Interpolation

**DOI:** 10.3390/plants13071050

**Published:** 2024-04-08

**Authors:** Juan Wang, Tingting Shi, Hui Wang, Meng Li, Xiaobo Zhang, Luqi Huang

**Affiliations:** 1 School of Pharmaceutical Sciences, Changchun University of Chinese Medicine, Changchun 130117, China; juanwangcacms@163.com; 2 State Key Laboratory for Quality Ensurance and Sustainable Use of Dao-di Herbs, National Resource Center for Chinese Materia Medica, China Academy of Chinese Medical Sciences, Beijing 100700, China; 3 China Academy of Chinese Medical Sciences, Beijing 100700, China

**Keywords:** wild *A. annua*, amount of *A. annua*, MaxEnt model, spatio-temporal kriging interpolation, geodetector

## Abstract

In order to determine the distribution area and amount of *Artemisia annua* Linn. (*A. annua*) in China, this study estimated the current amount of *A. annua* specimens based on the field survey sample data obtained from the Fourth National Census of Chinese Medicinal Resources. The amount was calculated using the maximum entropy model (MaxEnt model) and spatio-temporal kriging interpolation. The influencing factors affecting spatial variations in the amount were studied using geographic probes. The results indicated that the amount of *A. annua in* China was about 700 billion in 2019. *A. annua* was mainly distributed in the circular coastal belt of Shandong Peninsula, central Hebei, Tianjin, western Liaoning, and along the Yangtze River and in the middle and lower reaches of Jiangsu, Anhui, and the northern Chongqing provinces. The main factors affecting the amount are the precipitation in the wettest and the warmest seasons, the average annual precipitation, and the average temperature in the coldest and the driest seasons. The results show that the amount of *A. annua* is strongly influenced by precipitation and temperature.

## 1. Introduction

*Artemisia annua* Linn. (*A. annua*) is an annual herb in the genus Artemisia of the family Asteraceae [1]. The above-ground parts of this plant are dried and used to extract artemisinin [2]. Artemisinin has been approved by the World Health Organization (WHO) as the drug of choice in the treatment of malaria. It is also widely used in the production of proprietary Chinese medicines, for the treatment of malaria, antiviral, anti-inflammatory and anticancer. [3,4,5,6]. Therefore, it is of great significance to quantify the amount of *A. annua* in China and its spatial distribution. 

Current research on *A. annua* mainly focuses on suitable or potential distribution areas for this plant [7,8,9,10,11,12]. Wang D et al. [8] studied the distribution by using five models: classification tree analysis (CTA, rpart), random forest (RF, RandomForest), the maximum entropy model (MaxEnt) (dismo), artificial neural networks (ANNs, nnet), and support vector machines, and the results showed that *A. annua* is mainly distributed in mid-latitudes of western and central Europe, southeastern Asia, southeastern North America, and southeastern South America. Zhang Qin et al. [9] used the MaxEnt to predict areas in China that were ecologically suitable for *A. annua* based on 396 distribution points, and they found that the most suitable areas are mainly located in the eastern part of Sichuan province, the western part of Chongqing Municipality, Guizhou province, the Guangxi Zhuang Autonomous Region, the western part of Yunnan province, and the eastern part of Hubei province. Huang et al. [10] applied the Geographic Information System for Traditional Chinese Medicine (TCMGIS) to classify suitable areas in China, and they found that Youyang, Chongqing, and Guizhou are suitable areas. Fan Zhentao et al. [11] utilized GIS to classify ecologically suitable areas in Guangxi province into four levels. Zhang Xiaobo et al. [12] applied statistical analysis to study the relationship between the artemisinin content and the climatic factors and geographical distribution. However, the amount of *A. annua* in China has been understudied and remains unclear. In addition, the factors affecting the amount and distributions of this plant are also unclear.

In this paper, the amount of *A. annua* in China is studied based on data from the Fourth National Census of Traditional Chinese Medicine Resources. Suitable distribution areas were determined using the maximum entropy model (MaxEnt, Version 3.4.4, American Museum of Natural History, New York, NY, USA; Available from: http://biodiversityinformatics.amnh.org/open_source/maxent/ accessed on 10 September 2023), and the number of *A. annua* specimens in each area was investigated using the spatio-temporal kriging interpolation method. In addition, the influencing factors on the amount of *A. annua* were studied using GeoDetector (Beijing, China; Available from: http://www.geodetector.cn/ accessed on 20 September 2023). The MaxEnt model [13,14,15,16,17,18] takes existing geographical distribution information of a species and its environmental variables as constraints and employs specific algorithms to quantify the non-random relationship between the species’ distribution area and the environmental variables. The analytical result reflects the influencing factors with regard to species distribution and the degree to which the habitat is suitable for its growth. Moreover, the spatio-temporal kriging interpolation method [19,20,21,22,23,24] considers the trends and spatial correlations of the data in time and space, which prevents the loss of important information and improves the interpolation accuracy when faced with missing or anomalous data problems or limited field survey data. Finally, GeoDetector [25,26,27,28,29,30] measures the spatial heterogeneity, detects explanatory factors, and analyzes interactions between variables by calculating the q-statistic.

## 2. Results

### 2.1. Results of MaxEnt Model of A. annua’s Ecologically Suitable Areas

**Figure 1:**
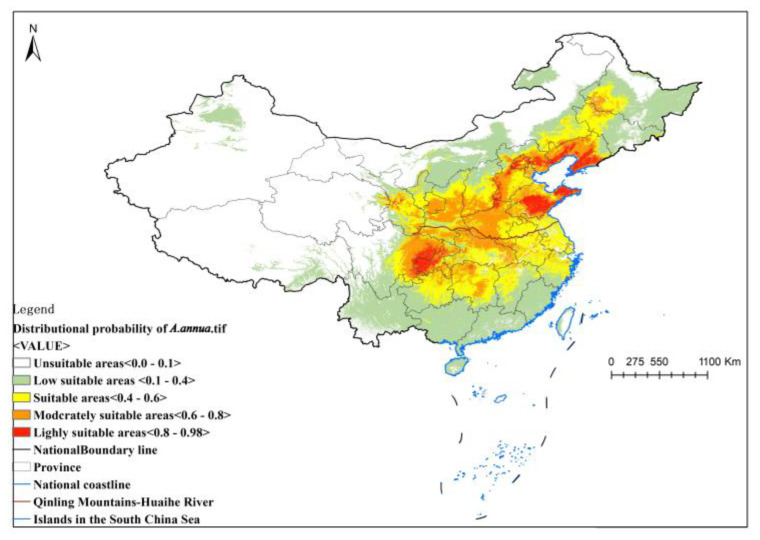
Ecologically suitable areas based on MaxEnt model results.

Based on the distribution probability results of the MaxEnt model, the distribution of suitable areas was reclassified in ArcMap 10.8 using manual classification. The results are shown in Figure 1.

The white areas on the map represent the unsuitable areas, with a distribution probability of 0–0.1; the green areas are poorly suitable areas, with a distribution probability of 0.1–0.4; the yellow areas are suitable areas, with a distribution probability of 0.4–0.6; the orange areas are the moderately suitable areas, with a distribution probability of 0.6–0.8; and the red areas are highly suitable areas, with a distribution probability of 0.8–0.98.

According to the figure, the suitable distribution area in China is large, and highly suitable areas are mainly distributed along the borders of Liaoning province, Hebei province, and Shandong province, as well as at the junction between Western Henan, Northwest Chongqing, and Sichuan province. Other suitable areas were mainly concentrated in the northwest of Jilin province and the west of Heilongjiang province, as well as Tianjin, Shanxi, Anhui, Hunan, Guizhou, Yunnan, Hubei, Jiangxi, Zhejiang, Jiangsu, and other provinces.

#### Accuracy Evaluation of the Results

**Figure 2:**
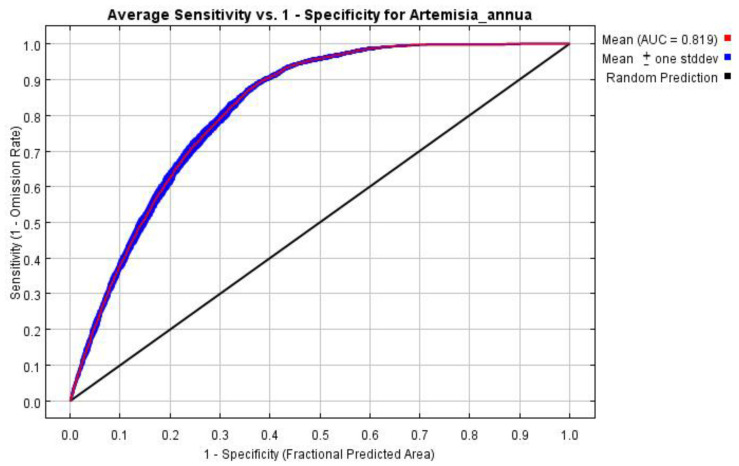
Validation of the ROC curve of the MaxEnt model for predicting the distribution of suitable areas.

In this study, the model was run 10 times, and the average value was taken to obtain the ROC curve (Figure 2). The average AUC value obtained during model training was 0.819, indicating good stability across different model replications. According to the evaluation criteria, the overall prediction accuracy of the model is good, indicating that the model predicts the distribution of suitable areas accurately.

As shown in Table 1, the main environmental factors affecting the distribution of *A. annua* in China were vegetation cover (FVC), the warmest seasonal mean temperature (BIO10), isothermality (BIO3), the precipitation during the wettest season (BIO16), and the coldest seasonal mean temperature (BIO11).

### 2.2. Spatio-Temporal Kriging Modeling Results for Estimating the Amount of A. annua

#### 2.2.1. Results of Sample Stratification

Table 2 shows the results of stratifying the sample data of the rational detector study. The q = 0.01 of the national sample data divided into southern and northern strata, taking the Qinling–Huaihe River as the dividing line, is greater than the results of the other strata. Therefore, after dividing the sample data into the northern layer and the southern layer, the difference between the layers was larger, which was more conducive to studying the amount of *A. annua* in China.

#### 2.2.2. Results of the Semi-Variate Function Study

Using spatio-temporal kriging interpolation to estimate the amount in July 2019 in China, the spatial and temporal variations after preprocessing were as follows.

**Figure 3:**
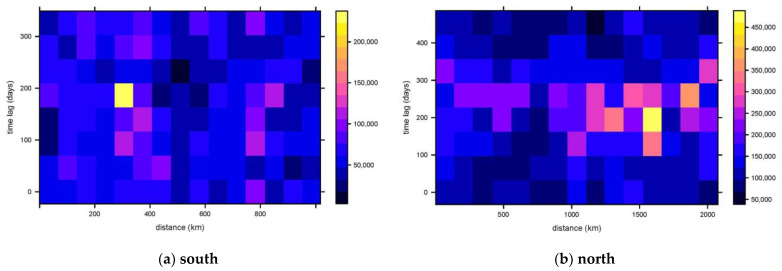
Spatial and temporal variability of the sample data of *A. annua* numbers. (**a**) Results of spatio-temporal variability in the southern layer of China. (**b**) Results of spatio-temporal variability in the northern layer of China.

The average distance in the spatial variation in the south is about 200 km based on empirical jugement (Figure 3a). This trend tends to stabilize after 200 km, indicating that the range of spatial autocorrelation is 200 km. Similarly, in the time dimension, the average tends to stabilize at about 80 days, indicating that the autocorrelation variation range in time is 80 days. The spatio-temporal anisotropy can thus be calculated as 75 km/month. In the north, the average spatial autocorrelation variation range is about 100 km. (Figure 3b) The average temporal autocorrelation range is about 100 days, so the spatio-temporal anisotropy in the north is 30 km/month.

**Figure 4:**
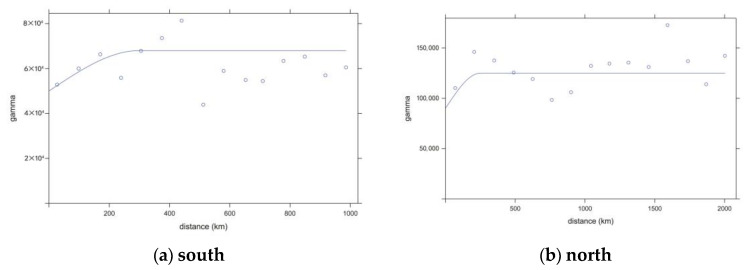
Semi-variogram functions and fitted curves for the southern and northern layers of China. (**a**) Semi-variance function and fitted curve for the southern layer of China. (**b**) Semi-variance function and fitted curve for the northern layer of China.

The semi-variance functions for the northern and southern regions were calculated as follows:

(1) $$\begin{array}{c} \gamma_{south}\left(H\right)=50,000+18,000\times \left(1.5\times \frac{H}{300}-0.5\times {\left(\frac{H}{300}\right)}^{3}\right) \end{array}$$

(2) $$\begin{array}{c} \gamma_{north}\left(H\right)=90,000+35,000\times \left(1.5\times \frac{H}{250}-0.5\times {\left(\frac{H}{250}\right)}^{3}\right) \end{array}$$

where *H* is the spatio-temporal distance; see Equations (8) and (9) for details. The constant term indicates the nugget effect, which reflects the systematic error; the coefficient term indicates the degree of correlation of the model in the spatial random field; and the distance term (the denominator in the exponential term) indicates the maximum distance of the spatio-temporal correlation. The spatio-temporal semi-variability function in both northern and southern regions is fitted by the joint spherical model. The results are shown in Figure 4.

#### 2.2.3. Model Accuracy Validation Results

The results of the model accuracy validation analysis after conducting leave-one-out cross-validation are shown in Table 3, with an average absolute error (MAE) of 293,000 and a root mean square error (RMSE) of 713,000 plants in the north, and an MAE of 142,000 and an RMSE of 302,000 plants in the south. Combining all errors in the north and south, the spatial model had a mean absolute error of 233,000 plants and a root mean square error of 585,000 plants.

#### 2.2.4. Number of *A. annua* Based on the Distribution of Suitable Areas

**Figure 5:**
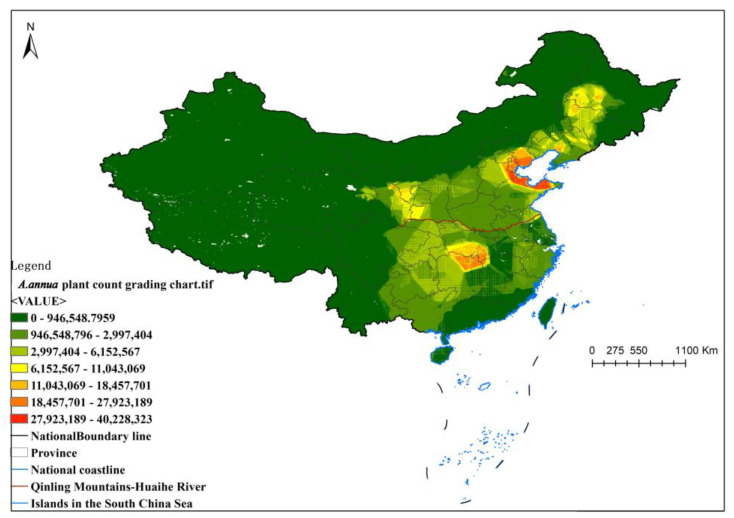
Results of *A. annua* plant count grading chart.

The final 1 km resolution distribution of *A. annua* is shown in Figure 5, which shows the areas with a probability of fitness greater than 0.1. The preliminary estimate of the total number of *A. annua* in China in September 2019 is more than 700 billion plants, of which there are more than 450 billion in the northern part of the country. In the north, these plants are mainly distributed in the Shandong Peninsula, the central part of Hebei province, Tianjin, and the western part of the coastal ring belt of Liaoning. To a lesser extent, these plants are distributed in the southern part of Heilongjiang province at the border of northern Jilin province, in western Gansu province at the border of Shaanxi province, and in Henan and Hebei province, as well as in Beijing. In the south, there are more than 250 billion plants, distributed along the Yangtze River and in the middle and lower reaches of the river basin, mainly in the northern areas of Jiangsu, Anhui, and Chongqing, and to a lesser extent in the border areas of Hubei, Hunan, and Jiangxi.

### 2.3. Analysis of the Drivers of Spatial Variation in the Distribution of the A. annua

The factor detection analysis conducted using Geodetector revealed the main driving factors affecting the amount of *A. annua* in each area, and the results are shown in Table 4. The factors that passed the *p* < 0.05 significance test and had a q-value greater than 0.05 are shown in Table 4. The results showed that the dominant factors affecting the distribution were the precipitation in the wettest season, the precipitation in the warmest season, the average annual precipitation, the average temperature of the coldest season, and the average temperature of the driest season.

## 3. Discussion

In this paper, we found that the MaxEnt model combined with spatio-temporal kriging interpolation was able to effectively estimate the amount of *A. annua* in China. The distribution areas were taken from the field survey of the Fourth National Census of Traditional Chinese Medicine Resources; the use of these data greatly enriched the distribution point data of *A. annua* in China in the process of MaxEnt modeling. In addition, the vegetation index factor was added as an environmental variable to evaluate the habitats of *A. annua*.

The results of MaxEnt modeling showed that *A. annua* has a wide range of suitable distribution areas in China, and the highly suitable areas are mainly in the junction of Liaoning province and Hebei province, Shandong province, western Henan province, northwestern Chongqing province, and Sichuan province. Other suitable areas are mainly concentrated in northwestern Jilin province, western Heilongjiang province, Tianjin, Shanxi, Ningxia, southwestern Gansu and Guizhou, Yunnan, Hunan, Anhui, Hubei, Jiangxi, Zhejiang, Jiangsu, and other provinces. The results of the ecological suitability zoning study in [9] showed that *A. annua* is mainly distributed in the southwest region of China, including in Chongqing, Sichuan, Yunnan, Guangxi, Yunnan, and Hunan provinces, which is consistent with the results of the study in this paper. The distribution points in this study total more than 4000, which are distributed in many provinces in China. The number of distribution points is larger than in other analyses [9], which indicates that the number of distribution points affects the predicted results of the MaxEnt model. The wider the spatial range of the distribution points and the richer the sample points are, the closer the results are to the actual situation.

The spatio-temporal kriging model estimated that the number of *A. annua* specimens in China is more than 700 billion. Previous studies [31,32,33] stopped at the estimation of distribution probabilities due to incomplete survey data or methodological limitations. Spatio-temporal geostatistical modeling allows for further quantification of species distributions with quantitative information, based on the principle that closer information is more relevant. Other studies [21,22,23,24] used regression kriging and combined regression relationships with geostatistics to obtain their estimation results. Meanwhile, this study found that the environmental variables affecting the abundance of *A. annua* had a nonlinear relationship with the amount, which was difficult to fit using traditional regression.

By using the GeoDetector, we found that the amount is mainly affected by the precipitation in the wettest season, the precipitation in the warmest season, and the average annual precipitation. Most of the precipitation in the wet season and in the warmest season occurred in the same period, indicating that a warm and humid environment with sufficient precipitation is favorable to the growth of *A. annua*, which is consistent with the biological characteristics of the plant, as it prefers high humidity and warm temperatures. The seeds of the plant do not have dormancy characteristics. During germination, seeds fall on the ground, and mature seeds can germinate and grow under natural conditions. In late September [34], new plants grow near the mature plants on the ground. During the dry and cold season, *A. annua* seeds gradually enter maturity and wilt, while the mature seeds begin their next life cycle. Additionally, the temperature of the season may affect the germination and growth of the seed, which ultimately affects the amount.

In summary, this paper is methodologically innovative, which lies in estimating the amount through a fusion of the MaxEnt model and spatiotemporal kriging interpolation. The derivation of distribution probability via ecological niche modeling serves as a foundational step for geostatistical spatial and quantitative estimation modeling using spatiotemporal kriging interpolation. It combines MaxEnt’s probability distribution abilities and the adjusted results of geostatistical interpolation to determine ecological significance. The stratified heterogeneity of the distribution was considered, and stratified modeling was used to avoid confusion in the results caused by the environmental differences between northern and southern China.

Compared with traditional geostatistical methods, ecologically, the totality of the plant amount is not continuous, and modeling is therefore highly dependent on the results of sample surveys. In this study, the combination of MaxEnt and spatio-temporal kriging was used to increase the number of *A. annua* in the areas with distribution probabilities less than 0.1 by increasing the number of sample points where the number is 0. Theoretically, the more intensive the sampling, the more accurate the obtained results, but the time and computational cost need to be taken into consideration. In addition, accurate SDMs (species distribution models) can also help in the estimation of the number of plants [35]. In addition, the uncertainty of the results comes from the process of modeling the spatio-temporal semi-variance function. The survey data samples are discrete, and there may be only one time transect for each survey datum in the same sample site, leading to a more fragmented spatio-temporal semi-variability function (Figure 3), which is difficult to be fitted using traditional spatio-temporal tools (spatio-temporal product sum function, spatio-temporal sum metric function, etc.) [36]. Therefore, in this study, spatio-temporal anisotropy is first derived as demonstrated in Figure 3, and then the empirical semi-variability function is computed and fitted using the spatio-temporal joint function and the metric model. Nevertheless, the uncertainty caused by the sample discretization remains and is still a source of errors.

## 4. Materials and Methods

### 4.1. Materials

#### 4.1.1. Study Scope and Species Distribution Points

**Figure 6:**
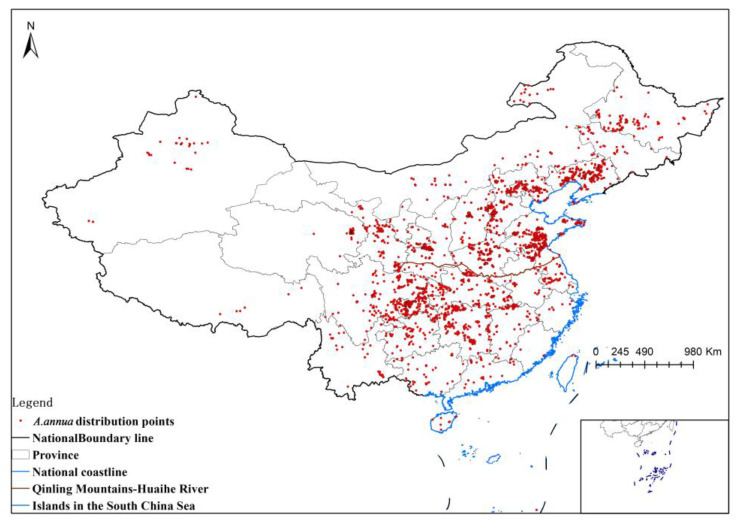
Distribution points of *A. annua*.

The distribution points were obtained from the field survey conducted by the Fourth Chinese Traditional Medicine Resources Census Survey Group during 2012–2020, with more than 3400 records; the data collected by the research group, with more than 530 records; and the data obtained from the GBIF website, counting more than 200 records. In total, there were more than 4100 distribution points, as plotted in Figure 6.

#### 4.1.2. Estimation of the Number of *A. annua* 1 km Survey Plots

**Figure 7:**
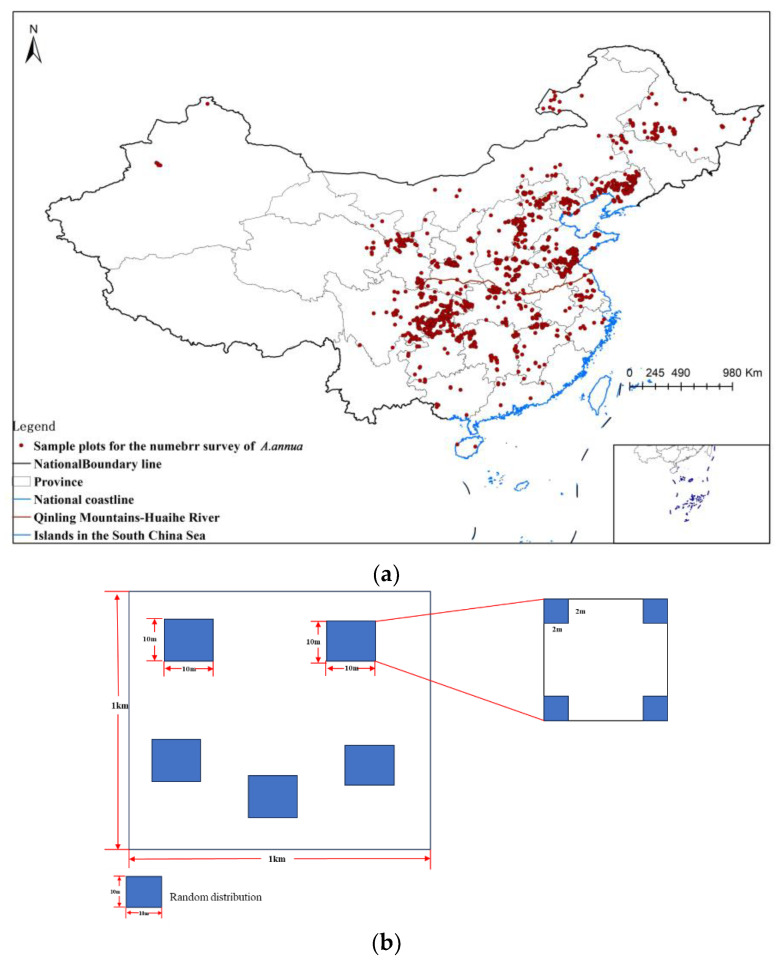
Distribution map of *A. annua* survey sample plots and schematic diagram of survey sample plots. (**a**) Map of *A. annua* survey sample sites; (**b**) Schematic diagram of the survey program for the *A. annua* sample plots.

To investigate the distribution of *A. annua*, we set up random sample plots and sample squares within the sample plots. The survey program set up 20 sample squares (2 m × 2 m) randomly in 1 km × 1 km sample plots to carry out the survey. The survey structure is shown in Figure 7b. A total of 1579 sample plots were collected during the period from 2012 to 2020 during the Fourth National Census of Traditional Chinese Medicine Resources. The results are shown in Figure 7a.

The formula for estimating the number in each 1 km survey square is as follows:

(3) $$\begin{array}{c} C=A\times \frac{1000\;\times \;1000\;\times \;F}{2\;\times \;2} \end{array}$$

where *C* is the number in the 1 km × 1 km plot; *A* is the mean number of plants in the sample, calculated as the total number of plants/number of samples; and *F* is the frequency of distribution, calculated as the number of samples where the species occurs/total number of samples surveyed.

**Figure 8:**
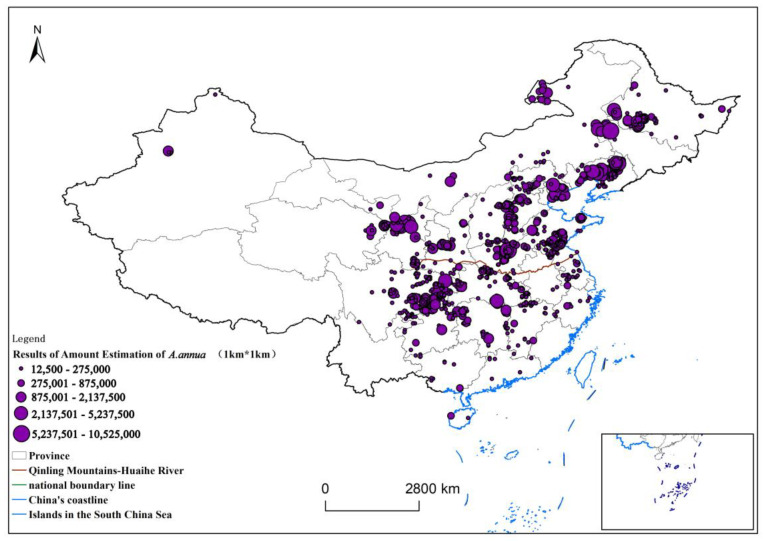
Grading map of *A. annua* amount at 1 km × 1 km scale.

Details on the number of *A. annua* specimens in the sample plot are shown in Table 5, and the results in the sample plot, graded using ArcMap 10.8, are shown in Figure 8.

#### 4.1.3. Selection and Preprocessing of Explanatory Variables

*A. annua* is an annual herb with a distribution influenced by climate and topography. The environmental variables used in the study were obtained from the Global Climate Database (http://www.worldclim.org/ (accessed on 3 March 2023)), and 19 bioclimatic variables were selected for ecological suitability zoning (Table 6). The modern climate data were from 1970 to 2000 with 30 arc-second resolution. The monthly [37] precipitation and monthly mean temperature data were obtained from a dataset of published papers in the journal Earth System Science Data (https://doi.org/10.5194/essd-11-1931-2019, (accessed on 10 March 2023). The topographic DEM elevation data were obtained from the SRTM topographic dataset released by NASA (http://srtm.csi.cgiar.org/ (accessed on 3 May 2023)) with a resolution of 1 km × 1 km. The surface analysis function of ArcMap 10.8 was used to extract the slope (1 km) and aspect.

The wild plants are mostly found on roadsides, in wastelands, on mountain slopes, and in forest margins; grasslands and forest steppes are also distribution points [38]. The vegetation index was used in the form of the environmental variable NDVI. The NDVI data were obtained from the National Tibetan Plateau Science Data Center (http://data.tpdc.ac.cn (accessed on 3 March 2023)), and the NDVI [39] dataset of the Chinese region at 250 m (2000–2022) can be accessed at https://data.tpdc.ac.cn/zh-hans/data/10535b0b-8502-4465-bc53-78bcf24387b3 (accessed on 3 March 2023). These data were resampled to 1 km. Vegetation cover data [40] were retrieved from the National Tibetan Plateau Science Data Center (http://data.tpdc.ac.cn (accessed on 3 March 2023)) and the China Regional 250 m Vegetation Cover Dataset (2000–2022) (https://data.tpdc.ac.cn/zh-hans/data/f3bae344-9d4b-4df6-82a0-81499c0f90f7 (accessed on 3 March 2023)), resampled to 1 km.

Vegetation type data were retrieved from the Resource and Environment Science and Data Center, Chinese Academy of Sciences (resdc.cn (accessed on 10 September 2023)).

Soil data were retrieved from the Resource and Environment Science and Data Center, Chinese Academy of Sciences (resdc.cn (accessed on 10 September 2023)).

Finally, Geodetector environmental factors were discretized, except for soil type and vegetation type.

### 4.2. Methods

**Figure 9:**
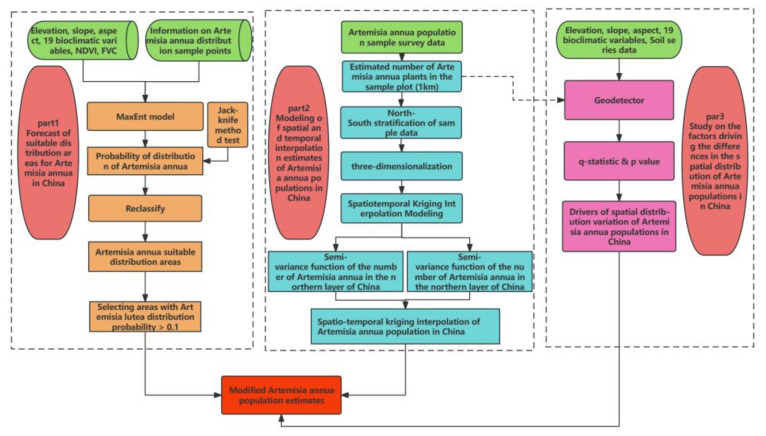
Technical roadmap for estimation of *A. annua* in China.

The workflow used in this research is provided in Figure 9.

In the first step, we collected data on the distribution points and environmental variables of *A. annua* and studied the distribution of suitable areas based on Maxent modeling. The result was the suitable distribution probabilities in each area.

In the second step, based on the sample data of the number from the sample plot survey, we constructed a spatio-temporal kriging interpolation model of sample stratification and a semi-variate function study and estimated the amount. In the final Maxent result, a 100 km × 100 km national systematic sampling was carried out, and the points with a distribution probability less than 0.1 were screened out as correction points. The number of plants in these points was set to 0, and the remaining points were used for the estimation of the final number of plants.

In the third step, the factors affecting the differences in the spatial distribution were revealed using Geodetector.

#### 4.2.1. Method for Studying the Ecologically Suitable Areas of *A. annua* in China

Ecologically suitable areas of *A. annua* in China were determined using the MaxEnt (maximum entropy) method. The theory of maximum entropy was first proposed in 1957 [31]. The Java MaxEnt model, which was developed from this theory, has become the most commonly used species distribution model (SDM). MaxEnt is a density estimation and species distribution prediction model based on the maximum entropy theory, which has the advantages of stable results and a short operation time. It is widely used in the analysis of plant and animal growth environments, pest early warning systems, habitat protection and the prediction of potential distribution areas [32,33]. It also has a wide range of applications in the prediction of potential ecologically suitable ranges of species, the effects of climate change on species distributions [41], and the conservation of endangered species [42].

The distribution points and environmental variables were saved as .csv data in the order of species name, longitude, and latitude and imported into MaxEnt 3.4.4 software for modeling. We randomly selected 75% of the distribution points as the training set and 25% as the test set. The maximum number of iterations was set to 1000, and the subsample method was selected to repeat the runs to create different test and validation sets. The calculation was repeated 10 times, the jack-knife method was employed to calculate the influence of environmental variables on the distribution, and the response curve of each environmental variable was drawn. The results were output in logistic form in .asc format, where the raster value was the probability of survival (*p*-value). The output results were converted to raster format in ArcMap 10.8.

The prediction results of the MaxEnt model were evaluated by the range of the AUC (area under curve), which is the area under the receiver operating characteristic (ROC) curve plotted with the specificity as the horizontal coordinate and the sensitivity as the vertical coordinate. The range of the AUC is 0–1, where the larger value means the further away from the random distribution and the better the prediction result. The value of the AUC is generally between 0 and 1, where an AUC value between 0 and 0.5 indicates that the model prediction failed; an AUC value between 0.6 and 0.7 indicates that the prediction effect is poor; an AUC value between 0.7 and 0.8 indicates that the prediction effect is general; an AUC value between 0.8 and 0.9 indicates that the prediction effect is satisfying; and an AUC value greater than 0.9 indicates that the prediction effect is good [43].

#### 4.2.2. Method for Estimating the of Amount *A. annua* in China

Data stratification: All collected sample data were stratified using the Geodetector method, which stratified the data according to the southern and northern regions of China, according to the month in which the data were collected and according to the season; analysis was conducted based on q-values and *p*-values.

The GeoDetector model uses the q-statistic to quantify the determinant powers of the influencing factors from spatio-temporal perspectives and the stratified heterogeneity of a dependent variable [25,44]. It is expressed as follows:

(4) $$\begin{array}{c} q=1-\frac{\sum_{h=1}^{L}N_{h}\sigma_{h}^{2}\;}{N\sigma^{2}}\; \end{array}$$

where *q* is the determining power of the environmental factor, which takes values ranging from 0 to 1 and represents the determinant power of the heterogeneity of the risk factor or target variable. h (h = 1, 2, …, L) denotes the spatial stratification of a single factorization X. N and $N_{h}$ are the numbers of units in the entire area and stratum h, respectively. $\sigma^{2}$ and $\sigma_{h}^{2}$ are the variances in the number of *A. annua* specimens in the entire area and stratum h, respectively.

Three-dimensionalization: The year at the time of collection of all sample points is transformed into a spatial z-value, which forms a three-dimensional coordinate with latitude and longitude. The anisotropy coefficient of year and spatial distance (unit: km/month) is equal to the ratio of the variance in the spatio-temporal semi-variance function of the samples in each stratum. Since *A. annua* is harvested during the flowering period as a raw material for artemisinin extraction, and it flowers from July to September [34,45], the month with the largest number of samples, July 2019, was chosen as the reference point and set as the time origin in this study.

(5) $$\begin{array}{c} k=\frac{{Range}_{spatial}}{{Range}_{temporal}}\; \end{array}$$

(6) $$\begin{array}{c} Z=k\times \left[\left(year-2019\right)\times 12+\left(month-7\right)\right] \end{array}$$

The modeling process of the spatio-temporal kriging interpolation method is as follows:

Assumption 1: In order to increase the sample information, all the count data from 2012 to 2020 are assumed to be the background value samples in China.

Assumption 2: The number of *A. annua* per km^2^ in China is a random variable and satisfies the second-order smooth assumption. The expectation of the number of plants at any point in space is the same, and the covariance of the number of plants at any two points is related to their distances rather than to their spatial locations.

The basic principle is as follows:

The number of plants in space is represented as a random variable $\;\left\{Z\left(s_{i}\right)\right.;i=1,2,\ldots n\}$, and the number of plants at any point can be represented as the sum of the weights of the surrounding sample points.

(7) $$\begin{array}{c} Z_{\left(S_{0}\right)}=\sum_{i=1}^{k}\lambda_{i}Z\left(s_{i}\right) \end{array}$$

where $Z(s_{0},t_{0})$ denotes the number of plants at the point to be measured, k denotes the number of sample points, $\lambda_{i}$ denotes the weighted contribution of sample point $s_{i}$ to the point to be measured at time $t_{i}$ and $Z(s_{i},t_{i})$ denotes the number of *A. annua* specimens in the measured sample.

A spatio-temporal semi-variogram is an index describing the spatial relationship characteristics of spatially random variables, and the empirical semi-variogram is calculated from two points in spatio-temporal distance H.

(8) $$\begin{array}{c} H=\sqrt{h^{2}+{\left(k.u\right)}^{2}} \end{array}$$

(9) $$\begin{array}{c} \gamma \left(H\right)=\frac{1}{2N\left(H\right)}\sum_{\alpha =1}^{N\left(H\right)}{\left[Z\left(S_{\alpha }t_{\alpha }\right)-Z\left(s_{\alpha }t_{\alpha }+H\right)\right]}^{2} \end{array}$$

where *H* is the spatio-temporal distance, *h* is the Euclidean spatial distance between two points, *u* is the time interval between two points, *k* is the spatio-temporal anisotropy, and *N*(*H*) represents the number of sample point pairs at each spatio-temporal distance. Under the constraint of unbiased optimality, the system of equations can be obtained:

(10) $$\left\{\begin{array}{l} \sum_{j=1}^{n}\lambda_{j}\gamma (s_{i},s_{j})+\mu =\gamma (s_{i},s_{0}),i=1,2,\ldots ,n\\ \sum_{j=1}^{n}\lambda_{j}=1 \end{array}\right.$$

By solving the equation, the weighted contribution of each sample point to the measurement point can be obtained and then substituted into Equation (7) to calculate the number of trees to be measured.

Since the spatial modeling assumes a certain constant expectation of strain counts across the country, it defies reality. Therefore, it is necessary to correct the strain count at each predicted site via ecological modeling. In this study, the MaxEnt model was used to estimate the probability of species distribution using covariates (19 climatic BIO variate factors, topographic factors of elevation, slope and slope direction data, monthly precipitation, monthly mean temperature, monthly minimum temperature, monthly maximum temperature, normalized vegetation index, and vegetation cover data) for the whole country in 1 km × 1 km plots.

In the final MaxEnt results, a 100 km × 100 km national systematic sampling was conducted to screen out the points with distribution probabilities less than 0.1 as correction points, and their amounts were set to 0 in the estimation of the final plant counts to ensure reasonable fit in the spatial prediction. At the same time, the points at which the distribution probability of the sample was less than 0.1 were removed and not involved in the calculation.

After fitting the model with sampled data, each data point is removed from the sampled dataset one at a time. Then, the leave-one-out dataset [46] is applied to the model to estimate the value at the removed point. The estimate is compared with the observed true value by calculating the experimental error. In this paper, the mean absolute error (MAE) and root-mean-square error (RMSE) are evaluated [47].

(11) $$\begin{array}{c} RMSE=\sqrt{\frac{1}{n}\sum_{i=1}^{n}{\left(X_{obs,i}-X_{pre,i}\right)}^{2}\;\;} \end{array}$$

where $X_{obs,i}$ denotes the observed value of *A. annua* at the i sample site, $X_{pre,i}$ represents the estimated value of the i sample site, and *n* is the number of observations. A smaller *RMSE* indicates a more precise interpolation model.

We also used the *MSE* to evaluate the validity of our methods. The *MSE* is usually used to describe the degree of change in the data and is expressed as

(12) $$\begin{array}{c} MSE=\frac{1}{n}\sum_{i=1}^{n}{\left(X_{obs,i}-X_{pre,i}\right)}^{2}\;\; \end{array}$$

where $X_{obs,i}$ denotes the observed value of *A. annua* at the i sample site, $X_{pre,i}$ represents the estimated value at sample site i, and *n* is the number of observations.

Geodetector q-statistics were used to explore the main influencing factors affecting the amount. The influencing role is expressed as the influence strength, as a q value of [0, 1], where a value closer to 0 indicates that the factor has a weaker influence, and closer to 1, the influence is stronger. In this paper, 19 climate variables, topographic factors (elevation, slope, slope direction), soil factors (soil type, soil Ph, soil clay content, soil sand content, soil effective water content), and vegetation type factors were selected as potential influencing factors to analyze the distribution based on geodetic probes. The number estimated from 1579 sample plots was used as the most dependent variable Y, and the extracted environmental variable data were used as the independent variables X. The above independent variables were discretized by removing the variables of soil type and vegetation type and then run in GeoDetector software (2015_Example) to assess the factors influencing the differences in the spatial distribution.

## 5. Conclusions

The main objective of this study is to estimate the amount of *A. annua* based on the reliable field survey sample data investigated by the Fourth National Census of Traditional Chinese Medicine Resources survey group. Since the survey was conducted during the 2012–2020 period and the spatial distribution of the surveyed areas were different across years, the inclusion of spatio-temporal kriging interpolation in this study was important for the estimation of the amount. Using the available distribution points of *A. annua*, MaxEnt modeling was used to study the distribution of suitable areas, and the estimated amount was corrected using spatio-temporal kriging interpolation. The main factors affecting the differences in the spatial distribution were explored using geographic probes. The results showed that the amount of *A. annua* is 700 billion in China, and the regions with the greatest distribution are the Shandong Peninsula, the central part of Hebei, Tianjin, the ring coastal belt of western Liaoning, Jiangsu, Anhui, and the northern part of Chongqing. Precipitation and mean annual precipitation in the warm and wet seasons and the average temperature in the dry and cold seasons are the main factors influencing the differences in the spatial distribution.

In this paper, we explored the time-discontinuous and spatially non-repeated field survey of the amount of *A. annua*, and the study of feasible methods for estimating the amount in contexts where discontinuous data do not obey a normal distribution is a major contribution of this paper.

## Figures and Tables

**Table 1:** Contribution and replacement importance of variables affecting the distribution of *A. annua* in China.

Variable	Percent Contribution	Permutation Importance
FVC	28.1	13.0
Bio 10	7.9	1.1
Bio 03	7.5	3.4
Bio 16	7.3	0.0
Bio 01	6.9	0.1
Bio 11	6.8	15.4
Bio 04	6.1	10.4
Bio 19	4.4	8.2
Elev	4.0	7.0
Bio 09	2.8	5.2
Bio 02	2.7	13.6
Bio 13	2.1	3.4
NDVI	1.6	1.0
tmx_2019_9	1.6	1.1
Bio 07	1.5	1.4
Bio 14	1.4	2.0
Slope	1.3	1.0
Bio 18	1.2	5.3
Bio 06	1.1	0.1
Bio 12	1.0	1.2
Bio 08	0.8	1.0
tmn_2019_9	0.6	0.9
Bio 15	0.4	0.6
tmp_2019_9	0.3	0
Bio 05	0.3	1.5
2019_9_pre	0.3	2.0
Aspect	0.1	0.2
Bio 17	0.0	0.1

**Table 2:** Differences in results following stratification.

Different Stratification	*q*	*p*
Northern and southern stratification of China	0.02	0.000
Stratification by season	0.01	0.030
Stratification by month	0.001	0.616

**Table 3:** Evaluation of spatial model uncertainty.

Title 1	MAE	RMSE
North	293,000	713,000
South	142,000	302,000
Total	233,000	585,000

**Table 4:** Geodetector factor results.

Variable	*q* Statistic	*p* Value
BIO 16	0.09	0.000
BIO 18	0.08	0.000
BIO 12	0.08	0.000
BIO 11	0.08	0.000
BIO 9	0.08	0.000
BIO 17	0.07	0.000
BIO 7	0.07	0.000
BIO 19	0.07	0.000
BIO 1	0.07	0.000
Type of vegetation	0.07	0.000
BIO 8	0.07	0.000
BIO 6	0.06	0.000
BIO 4	0.06	0.000
BIO 10	0.06	0.000
BIO 13	0.06	0.000
BIO 14	0.06	0.000
Type of soil	0.05	0.000
BIO 5	0.05	0.000
Organic carbon content	0.04	0.000
BIO 15	0.04	0.000
pH value of soil	0.04	0.000
BIO 2	0.04	0.000
Slope	0.03	0.000
BIO 3	0.03	0.000
Clay content of soil	0.02	0.000
elevation	0.02	0.000
Sand content of the soil	0.02	0.000
aspect	0.00	0.250
Effective water content of soil	0.00	0.640

**Table 5:** Sample amount of *A. annua* at the sample plot (1 km × 1 km) scale.

Year	Sample Sites	Number of *A. annua* Specimens (Millions)
2012	166	44.225
2013	222	49.6375
2014	159	46.95
2015	132	27.7875
2016	15	14.7375
2017	39	7.4125
2018	347	109.125
2019	472	128.9
2020	27	12.325

**Table 6:** Detailed explanation of variables.

Variables	Description
BIO1	Annual Mean Temperature
BIO2	Mean Diurnal Range (Mean of monthly temperature (max temp–min temp))
BIO3	Isothermality (BIO2/BIO7) (×100)
BIO4	Temperature Seasonality (standard deviation × 100)
BIO5	Max Temperature of Warmest Month
BIO6	Min Temperature of Coldest Month
BIO7	Temperature Annual Range (BIO5–BIO6)
BIO8	Mean Temperature of Wettest Quarter
BIO9	Mean Temperature of Driest Quarter
BIO10	Mean Temperature of Warmest Quarter
BIO11	Mean Temperature of Coldest Quarter
BIO12	Annual Precipitation
BIO13	Precipitation of Wettest Month
BIO14	Precipitation of Driest Month
BIO15	Precipitation Seasonality (Coefficient of Variation)
BIO16	Precipitation of Wettest Quarter
BIO17	Precipitation of Driest Quarter
BIO18	Precipitation of Warmest Quarter
BIO19	Precipitation of Coldest Quarter
elev	Elevation
Per	Precipitation
tmin	Monthly Minimum Temperature
tmax	Monthly Maximum Temperature
NDVI	Normalized Difference Vegetation Index
FVC	Fraction Vegetation Coverage

## Data Availability

Data are contained within the article.
